# Cumulative live birth rates after IVF/ICSI cycles with sperm prepared by density gradient centrifugation vs. swim-up: a retrospective study using a propensity score-matching analysis

**DOI:** 10.1186/s12958-022-00933-2

**Published:** 2022-03-31

**Authors:** Meng Rao, Li Tang, Longda Wang, Mengxiang Chen, Gaofeng Yan, Shuhua Zhao

**Affiliations:** Department of Reproduction and Genetics, First Affiliated Hospital of Kunming Medical University, Kunming, 650032 Yunnan Province China

**Keywords:** Sperm preparation, Density gradient centrifugation, Swim-up, IVF, Cumulative live birth rate

## Abstract

**Background:**

Density gradient centrifugation (DGC) and swim-up (SU) are the two most widely used sperm preparation methods for in vitro fertilization (IVF) and intracytoplasmic sperm injection (ICSI). However, existing comparisons of IVF/ICSI outcomes following these sperm preparation methods are insufficient and controversial.

**Methods:**

This retrospective study included all first autologous IVF and ICSI cycles performed between March 1, 2016, and December 31, 2020 in a single university-based center. A total of 3608 cycles were matched between DGC and SU using propensity score (PS) matching for potential confounding factors at a ratio of 1:1. The primary outcome was the cumulative live birth rate (cLBR) per aspiration.

**Results:**

PS matching provided 719 cycles after DGC and 719 cycles after SU. After adjusting for confounders, the recovery rate, progressive motility rate after sperm preparation, fertilization rate, good-quality embryo rate, and blastocyst formation rate were similar between the DGC and SU groups. The cLBR (odds ratio [OR] = 1.143, 95% confidence interval [CI]: 0.893–1.461) and LBR per transfer (OR = 1.082, 95% CI: 0.896–1.307) were also not significantly different between the groups. Furthermore, no significant differences were found in all of the laboratory and clinical outcomes following conventional IVF or ICSI cycles between the two groups. However, a significantly higher fertilization rate (β = 0.074, 95% CI: 0.008–0.140) was observed when using poor-quality sperm in the DGC group than in the SU group.

**Conclusions:**

Sperm preparation using DGC and SU separately resulted in similar IVF/ICSI outcomes. Further studies are warranted to compare the effects of these methods on IVF/ICSI outcomes when using sperm from subgroups of different quality.

**Supplementary Information:**

The online version contains supplementary material available at 10.1186/s12958-022-00933-2.

## Background

The prevalence of infertility is approximately 12–15% globally [1]. Assisted reproductive technologies (ARTs) have helped millions of couples deliver their own babies [2, 3]. However, the success rates of ARTs are still unsatisfactory. Only approximately one-third of all in vitro fertilization (IVF) and intracytoplasmic sperm injection (ICSI) cycles result in a live birth [2, 3]. Semen parameters affect both embryo quality and IVF/ICSI outcomes, and the relative contribution of the sperm to a successful live birth can be hypothesized to be 10–15% [4, 5]. As sperm show high inter-sample heterogeneity, sperm preparation to yield high-quality sperm for fertilization is crucial for improving IVF/ICSI outcomes.

Swim-up (SU) and density gradient centrifugation (DGC) are the two most widely used sperm preparation methods for ART. Both methods are recommended by the World Health Organization (WHO) for recovering motile spermatozoa with morphologically normal forms and free of seminal plasma, debris, non-germ cells, and dead spermatozoa [6]. Although the efficiencies of these two methods have been compared since the 1980s, most of these studies have focused on semen parameters such as the recovery rate, concentration, progressive motility rate (PR), morphology, and sperm DNA fragmentation (SDF) of the recovered sperm. Most of these studies report a higher recovery rate following DGC but a higher PR following SU [7–15]. However, the results regarding the SDF [7, 16–23] and normal morphology rate [7, 12–15, 20, 21, 24–30] remain contradictory. In addition, only a few studies have evaluated other parameters that may affect IVF/ICSI outcomes. Some of these have reported that sperm prepared by DGC (vs. SU) have better acrosome function, capacitation, and hyper-activation [8, 28, 31], whereas others have found that sperm prepared by SU (vs. DGC) have fewer vacuoles in the heads [7, 23].

The comparisons of ART outcomes after DGC and SU are insufficient due to the limited number of studies and limited sample sizes in the published studies. A Cochrane meta-analysis in 2019 included four randomized controlled trials comprising 370 participants for comparison of the clinical pregnancy rates (CPRs) after artificial insemination with the husband’s sample following DGC and SU [32]. No significant difference was found in the CPR between the SU and DGC groups (CPR 22% vs. 24%; odds ratio [OR] = 0.83, 95% confidence interval [CI]: 0.51–1.35; I^2^ = 71%). However, the evidence level was very low because of high heterogeneity and the limited number of participants. The effects of these sperm preparation methods on the IVF/ICSI outcomes have also been found to be inconsistent. For example, Van der Zwalmen et al. [29] reported a higher ongoing pregnancy rate after IVF cycles with sperm prepared by DGC than after IVF cycles with sperm prepared by SU. In contrast, Palini [33] found that the blastulation rates per fertilized oocytes (41.7% vs. 58.5%, *p* = 0.009), blastulation rates per D3 embryos (46.1% vs. 63.7%, *p* = 0.045), and pregnancy rates (25.8% vs. 41.9%, *p* = 0.045) were higher after ICSI in the direct micro SU group than in the DGC group. Other researchers did not find any significant difference in IVF/ICSI outcomes between the SU and DGC groups [25, 26, 34]. These inconsistent results may be due to the differences in SU and DGC procedures used in different studies and the limited sample sizes.

To compare the differences in IVF/ICSI outcomes following sperm preparation by DGC vs. SU, we retrospectively analyzed the outcomes of first IVF/ICSI cycles with sperm prepared by DGC or SU. By propensity score (PS) matching, 719 cycles with sperm prepared by DGC and 719 with sperm prepared by SU were compared for the cumulative live birth rate (cLBR) per aspiration, and the recovery rate, PR after sperm preparation, fertilization rate, good-quality embryo rate, blastocyst formation rate, LBR per transfer. We found no significant difference in the cLBR between the DGC and SU groups or between patient subgroups stratified by the fertilization method (IVF vs. ICSI) and sperm quality (normal vs. poor).

## Materials and methods

### Study population and design

This retrospective study was conducted in the reproductive center of the First Affiliated Hospital of Kunming Medical University. First IVF and ICSI cycles performed between March 1, 2016, and December 31, 2020 were assessed for inclusion. Only cycles that resulted in at least one live birth after a fresh embryo transfer (ET) or consecutive frozen ETs or cycles that failed to give a live birth after all available embryos had been transferred were included. The other inclusion criteria were autologous sperm and oocytes, a female age ≤ 40 years, and sperm prepared by either DGC or SU. Cycles were excluded if (i) the female partner had been diagnosed with recurrent pregnancy loss, uterine malformation, and/or adenomyosis; (ii) male or female chromosomal abnormality was reported; or (iii) surgical sperm or frozen–thawed testicular sperm were used. A total of 3608 cycles were included for further analysis.

### Semen sample collection, evaluation, and preparation

Semen samples were collected on the day of oocyte aspiration following 2–7 days of ejaculatory abstinence. After fluidification, the samples were analyzed according to the WHO guidelines (2010) [6]. A sample was considered to be of poor quality if it had one or more parameters below the reference thresholds (concentration < 15 × 10^6^/mL, total spermatozoa < 39 × 10^6^/mL, and/or PR < 32%). The semen samples were then prepared by SU or DGC, and the recovered sperm were evaluated again by the same technician who had analyzed the sperm samples before preparation.

SU: Briefly, semen samples were first transferred to individual 15-mL centrifuge tubes. Then, 2 mL of G-IVF plus (Vitrolife Sweden AB, V. Frölunda, Sweden) was gently layered above the semen. The tubes were inclined at a 45° angle and incubated at 37 °C for 40–60 min. After incubation, 1.5 mL of the supernatant was transferred to a new tube and centrifuged at 300×*g* for 6 min. The resulting supernatant was discarded, and the sperm pellet was resuspended in 2 mL of G-IVF plus, followed by further centrifugation at 300×*g* for 6 min. The resulting supernatant was discarded, and the sperm pellet was resuspended in 0.5–1 mL of the culture medium for further use.

DGC: A two-layer gradient was prepared with 1.5 mL each of 45 and 90% SpermGrad (Vitrolife Sweden AB, V. Frölunda, Sweden) in 15-mL centrifuge tubes. The semen samples were transferred to the top of the gradient in individual tubes and centrifuged at 300×*g* for 20 min. The resulting supernatant was discarded, and the pellet was resuspended in 2 mL of G-IVF plus, followed by centrifugation at 300×*g* for 6 min. The resulting supernatant was again discarded, and the sperm pellet was resuspended in 0.5–1 mL of the culture medium for further use.

### Ovarian stimulation, oocyte aspiration, fertilization, and ET

Different protocols were used for ovarian stimulation according to the woman’s condition. The details of these protocols, including the use of gonadotropin-releasing hormone agonist or antagonist or other protocols (mild stimulation and progestin-primed ovarian stimulation), have been described in our previous study [35]. After retrieval, oocytes were fertilized via conventional IVF or ICSI. The insemination, evaluation of the fertilization status, Day 3 embryo grading, and blastocyst scoring were performed as described in our previous study [36]. At most, three Day 3 embryos or two blastocysts were transferred. The remaining available embryos were frozen using a vitrification kit (KITAZATO BioPharma, Shizuoka, Japan) and then thawed for a frozen ET if the previous ET cycle had failed.

### Study variables and outcomes

Baseline demographic data of each patient were exported from the ART database of our center. These included age, body mass index (BMI), semen parameters, and smoking and drinking statuses of the male partner; age, BMI, serum concentration of anti-Mullerian hormone (AMH), and type of infertility of the female partner; and IVF characteristics including the ovarian stimulation protocol, retrieved oocytes, and fertilization method (IVF/ICSI).

The primary outcome in this study was the cLBR per aspiration. The secondary outcomes were the recovery rate, PR after sperm preparation, fertilization rate, good-quality embryo rate, blastocyst formation rate, and LBR per transfer. The recovery rate was calculated as the total number of spermatozoa after sperm preparation divided by the total number of spermatozoa before sperm preparation. The fertilization rate was calculated as the number of normally fertilized oocytes (two pronuclei) divided by the total number of oocytes inseminated (IVF cycle) or the total number of oocytes injected (ICSI cycle). The good-quality embryo rate was calculated as the number of Grade I and II embryos divided by the total number of Day 3 embryos evaluated. The blastocyst formation rate was calculated as the number of blastocysts divided by the number of embryos that underwent blastocyst culture. The cLBR (%) was calculated as the number of oocyte aspiration cycles resulting in at least one live birth divided by the number of aspiration cycles × 100. The LBR was defined as the number of ET cycles resulting in at least one live birth divided by the number of ET cycles.

### Statistical analysis

The initial analyses of demographic data from the DGC (*n* = 2418) and SU (*n* = 1190) groups showed that the mean numbers of retrieved oocytes were 14.2 and 6.8, respectively. Considering that the oocyte number had a significant effect on the cLBR [37] and that the study time span was approximately 5 years, we used PS matching without replacement to match the number of retrieved oocytes and the year of oocyte aspiration between the DGC and SU groups, with a 0.2 standard deviation caliper width and a 1:1 ratio.

Continuous variables with normal distribution are presented as means (standard deviations), whereas those with skewed distribution are presented as medians (interquartile ranges). The significance of differences in the demographic and clinical data between the DGC and SU groups was tested using an analysis of variance (continuous variables with normal distribution), a Mann–Whitney U test (continuous variables with skewed distribution), or a chi-square test (categorical variables).

Generalized linear models were used to evaluate the associations of sperm preparation methods with the recovery rate, PR after sperm preparation, fertilization rate, good-quality embryo rate, and blastocyst formation rate. For the recovery rate and PR after sperm preparation, the models were adjusted for male age, total sperm, and PR before preparation. For the fertilization rate, good-quality embryo rate, and blastocyst formation rate, the models were adjusted for male age, sperm quality, female age, female infertility type, ovarian stimulation protocol, number of retrieved oocytes, IVF/ICSI, and AMH. A multiple logistic regression analysis was performed to investigate the relationships of sperm preparation methods with the cLBR and LBR per ET. For the cLBR, the model was adjusted for male age, sperm quality, female age, female infertility type, ovarian stimulation protocol, number of retrieved oocytes, IVF/ICSI, and AMH. For the LBR per ET, the model was adjusted for male age, sperm quality, female age, female infertility type, ovarian stimulation protocol, number of retrieved oocytes, IVF/ICSI, AMH, fresh or frozen ET, number of transferred embryos, and the stage of transferred embryos (Day 3 embryo or blastocyst).

Previous studies have suggested that sperm quality influences the outcomes of sperm prepared by SU and DGC. Thus, we performed subgroup analyses based on sperm quality as well as male age, which is reported to be associated with sperm quality. We also compared the effects of DGC and SU on the outcomes of conventional IVF and ICSI cycles, respectively, as the sperm selection mechanisms are distinct for these two insemination methods.

All tests were two-tailed, and *p* < 0.05 was considered to be statistically significant. SPSS 25.0 (SPSS, Inc., Chicago, IL) was used for the data analyses.

## Results

**Fig. 1:**
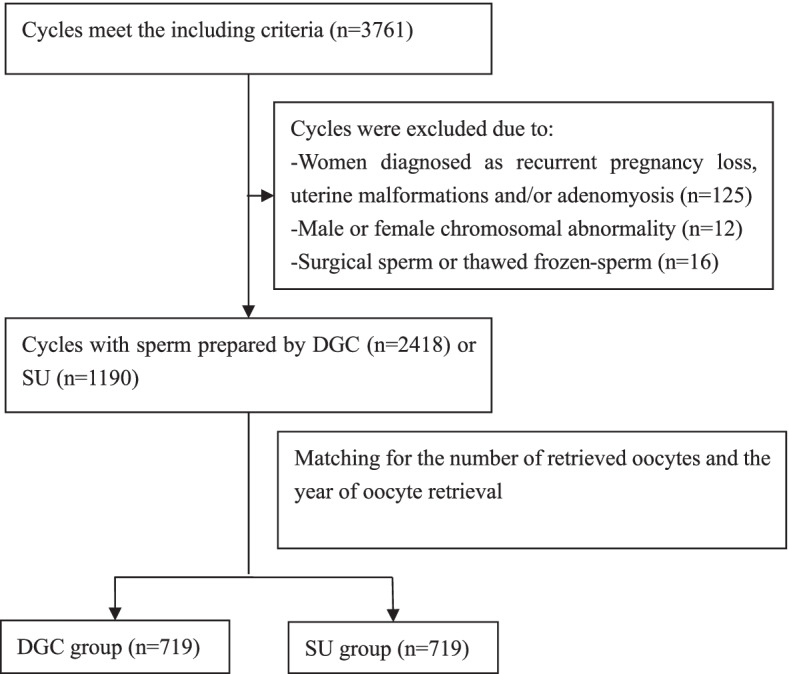
Flowchart of participant selection. SU, swim-up; DGC, density gradient centrifugation

Based on the inclusion and exclusion criteria, 3608 couples undergoing their first autologous IVF/ICSI cycles were included in this study. After PS matching, 719 cycles for each group were obtained (Fig. 1). The demographic characteristics of the participants are shown in Table 1. The number of retrieved oocytes was comparable between the DGC and SU groups. The male age, BMI, and smoking and drinking statuses were similar between the two groups. The semen parameters, including the total sperm number, concentration, and PR, were better in the SU group than in the DGC group. Consequently, the proportion of men with poor semen parameters was significantly lower in the SU group. The female age, BMI, and infertility type were not significantly different between the groups. Compared with the DGC group, more women in the SU group had a low AMH level (< 1.5 ng/mL) and underwent ovarian stimulation by protocols other than the use of gonadotropin-releasing hormone agonist or antagonist. The proportion of conventional IVF or ICSI cycles was similar between the two groups.

**Table 1:** Characteristics of included participants

Variables	DGC	SU	P
No. of cycles	719	719	
**Male**			
Age, years, mean (SD)	33.5 (5.5)	33.7 (5.4)	0.509
BMI, kg/m^2^, mean (SD)	24.1 (3.4)	24.3 (3.4)	0.341
Total sperm, ×10^6^, median (IQR)	87.5 (55.1–136.0)	105.0 (70.0–156.0)	< 0.001
Concentration, ×10^6^/mL,median (IQR)	35.0 (24.0–50.0)	43.0 (33.0–57.0)	< 0.001
PR, %, median (IQR)	45.0 (38.0–55.0)	50.0 (41.0–58.0)	< 0.001
Semen quality, % (n)			
Normal	73.2 (526)	85 (611)	< 0.001
Poor	26.8 (193)	15.0 (108)	
Smoking, % (n)			
Never	46.7 (336)	44.5 (320)	0.126
Current	22.3 (160)	19.6 (141)	
Former	31.0 (223)	35.9 (258)	
Drinking, % (n)			
Never	46.3 (333)	44.8 (322)	0.787
Current	23.1 (166)	24.5 (176)	
Former	30.6 (220)	30.7 (221)	
**Female**			
Age, years, mean (SD)	31.5 (4.3)	31.8 (4.4)	0.111
BMI, kg/m^2^, mean (SD)	22.3 (3.3)	22.4 (3.4)	0.832
AMH, ng/mL, % (n)			
< 1.5	20.0 (144)	25.6 (184)	0.012
≥ 1.5	80.0 (575)	74.4 (535)	
Type of infertility, % (n)			
Primary	45.6 (328)	43.8 (315)	0.491
Secondary	54.4 (391)	56.2 (404)	
Ovarian stimulation protocol, % (n)			
Agonist protocol	54.2 (390)	47.1 (339)	0.005
Antagonist protocol	34.1 (245)	36.3 (261)	
Other protocols	11.7 (84)	16.5 (119)	
Retrieved oocytes, median (IQR)	9.0 (6.0–12.0)	9.0 (5.0–12.0)	0.334
IVF/ICSI, % (n)			
IVF	87.3 (628)	86.5 (622)	0.639
ICSI	12.7 (91)	13.5 (97)	

*DGC* Density gradient centrifugation, *SU* Swim-up, *SD* Standard deviation, *IQR* interquartile range, *BMI* Body mass index, *PR* Progressive motility rate, *AMH* Anti-Mullerian hormone, *IVF* In vitro fertilization, *ICSI* Intracytoplasmic sperm injection

The cLBRs were 62.7 and 58.7% in the DGC and SU groups, respectively (Table 2). After adjusting for confounders, no significant difference was observed in the cLBR (OR = 1.143, 95% CI: 0.893–1.461) and the LBR per transfer (OR = 1.082, 95% CI: 0.896–1.307) between the groups (Table 2). The laboratory outcomes, including the sperm recovery rate, PR after sperm preparation, fertilization rate, good quality embryo rate, and blastocyst formation rate, were similar between the groups (Table 2).

**Table 2:** Comparisons of outcomes between cycles preparing sperm with GC and SU

Outcomes	DGC	SU	Adjusted β or OR (95%CI)	P
Recovery rate, %	21.0 (13.3–29.7)	21.2 (14.1–29.6)	−0.018 (− 0.064–0.028)^a^	0.439
PR after preparation, %	85 (81–87)	87 (84–88)	−0.002 (− 0.011–0.007)^a^	0.624
Fertilization rate, %	71.0 (25.5)	69.8 (25.5)	0.017 (−0.020–0.055)^a^	0.363
Good-quality embryo rate, %	48.0 (30.5)	47.3 (29.9)	0.025 (−0.042–0.092)^a^	0.466
Blastocyst formation rate, %	52.2 (25.2)	51.3 (25.6)	−0.001 (− 0.079–0.077)^a^	0.978
cLBR, % (n/n)	62.7 (451/719)	58.7 (422/719)	1.143 (0.893–1.461)^b^	0.289
LBR per transfer, % (n/n)	45.5 (457/1004)	42.9 (427/995)	1.082 (0.896–1.307)^b^	0.413

*DGC* Density gradient centrifugation, *SU* Swim-up, *OR* Odd ratio, *PR* Progressive motility rate, *cLBR* Cumulative live birth rate

^a^Adjusted β (95%CI), DGC vs. SU

^b^Adjusted OR (95%CI), DGC vs. SU

There were 628 IVF and 91 ICSI cycles in the DGC group, and 622 IVF and 97 ICSI cycles in the SU group. All of the clinical and laboratory outcomes were comparable between the two groups for both patients undergoing conventional IVF and those undergoing ICSI (Table 3).

**Table 3:** Comparisons of outcomes between cycles preparing sperm with DGC and SU following IVF and ICSI treatments

Outcomes	IVF	ICSI
No. of cycles		
DGC	628	91
SU	622	97
Recovery rate, %	−0.028 (− 0.074–0.18)^a^	− 0.040 (− 0.229–0.149)^a^
PR after preparation, %	− 0.006 (− 0.014–0.001)^a^	−0.002 (− 0.048–0.043)^a^
Fertilization rate, %	0.004 (− 0.036–0.045)^a^	0.086 (− 0.011–0.184)^a^
Good-quality embryo rate, %	0.020 (− 0.051–0.090)^a^	0.072 (− 0.132–0.276)^a^
Blastocyst formation rate, %	0.000 (− 0.082–0.082)^a^	0.043 (− 0.220–0.306)^a^
cLBR, % (n/n)	1.100 (0.843–1.434)^b^	1.389 (0.710–2.716)^b^
LBR per transfer, % (n/n)	1.086 (0.887–1.330)^b^	1.064 (0.613–1.845)^b^

*IVF* In vitro fertilization, *ICSI* Intracytoplasmic sperm injection, *DGC* Density gradient centrifugation, *SU* Swim-up, *PR* Progressive motility rate, *cLBR* Cumulative live birth rate

^a^Adjusted β (95%CI), DGC vs. SU

^b^Adjusted OR (95%CI), DGC vs. SU

In total, 193 men had poor semen parameters in the DGC group compared with 118 men in the SU group (Table 4). For cycles with poor-quality sperm, the fertilization rate was significantly higher in the DGC group than in the SU group (β = 0.074, 95% CI: 0.008–0.140) (Table 4). There was a trend, although not significant, toward a higher cLBR for cycles with poor-quality sperm in the DGC group (OR = 1.539, 95% CI: 0.825–2.823). For cycles with normal-quality sperm, no significant difference was observed in any of the outcomes between the DGC and SU groups (Table 4).

**Table 4:** Comparisons of outcomes between GC and SU in men with normal and poor sperm parameters

Outcomes	Normal	Poor
No. of cycles		
DGC	526	193
SU	611	118
Recovery rate, %	− 0.043 (− 0.089–0.002)^a^	0.025 (− 0.009–0.058)^a^
PR after preparation, %	−0.005 (− 0.014–0.003)^a^	1.858 (− 0.543–4.260)^a^
Fertilization rate, %	0.003 (− 0.037–0.043)^a^	0.074 (0.008–0.140)^a^
Good-quality embryo rate, %	0.011 (−0.062–0.085)^a^	0.028 (− 0.052–0.108)^a^
Blastocyst formation rate, %	−0.013 (− 0.101–0.074)^a^	0.010 (− 0.089–0.110)^a^
cLBR, % (n/n)	0.953 (0.644–1.411)^b^	1.539 (0.825–2.873)^b^
LBR per transfer, % (n/n)	1.114 (0.904–1.373)^b^	0.910 (0.575–1.442)^b^

*DGC* Density gradient centrifugation, *SU* Swim-up, *PR* Progressive motility rate, *cLBR* Cumulative live birth rate

^a^Adjusted β (95%CI), DGC vs. SU

^b^Adjusted OR (95%CI), DGC vs. SU

As a high male age is associated with poor semen parameters and higher SDF [38], we compared the effects of DGC and SU on the outcomes of cycles when using sperm from different male age groups. We found that all of the outcomes were similar between the DGC and SU groups for all age groups (Additional Table 1).

## Discussion

To the best of our knowledge, this is the first study to compare the cLBR after IVF/ICSI cycles with sperm prepared by DGC vs. SU. The cLBR, a key indicator of the effect of IVF/ICSI, is superior to the CPR and LBR per transfer for evaluating the effect of male factors, as it takes into consideration the overall embryo quality rather than the quality of just one or two embryos that affect the CPR or LBR per transfer.

Our results showed no difference in the cLBR between the DGC and SU groups or between subgroups stratified by the fertilization method (IVF vs. ICSI) and sperm quality (normal vs. poor). These results are consistent with previous reports. For instance, Hammadeh et al. [26] found that the pregnancy rate and implantation rate after conventional IVF cycles with sperm prepared by DGC (*n* = 60) were similar to those with sperm prepared by SU (*n* = 60). Soderlund and Lundin [34] also found no significant differences in pregnancy and the ongoing pregnancy rate between cycles with sperm prepared by DGC (*n* = 63) and those with sperm prepared by SU (*n* = 88). Borges [25] reported that the implantation, pregnancy, and miscarriage rates after intracytoplasmic morphologically selected sperm injection were not statistically different between the SU (*n* = 44) and DGC (*n* = 26) groups. In contrast, another study by Van Der Zwalmen et al. [29] published in 1991 reported significantly higher ongoing pregnancy and delivery rates after conventional IVF cycles with sperm prepared by DGC (*n* = 111) than after those with sperm prepared by SU (*n* = 185). However, the authors did not provide the basic demographic characteristics of the included patients and did not adjust for potential confounders.

In the current study, the fertilization rate from the sperm of men with poor semen parameters was significantly higher in the DGC group than in the SU group (Table 4). This is consistent with a previous report by Van Der Zwalmen [29]. This finding may be explained by DGC’s selection of sperm with good capacitation and acrosome reaction abilities, which are key indicators of the ability of sperm to fertilize an egg [8, 28, 31]. That is, the DGC selects morphologically normal sperm cells, especially sperm with normal heads, based on their specific density. The sperm recovered by DGC thus have better acrosome function, capacitation, and sperm hyperactivation than those recovered by SU [8, 28, 31]. The resulting higher fertilization rate following DGC increases the number of available embryos and results in a better cLBR relative to SU. Indeed, we found a trend, although not significant, toward a higher cLBR in cycles with sperm prepared by DGC from men with poor semen parameters. These findings further confirm the WHO’s fifth recommendation that DGC is a suitable preparation method for sperm from samples with poor semen parameters.

In sperm selection during natural conception, tens of millions of spermatozoa are selected at several sites in the female genital tract, namely, the (i) cervix, (ii) uterus, (iii) uterotubal junction, (iv) oviduct, (v) cumulus oophorus, and (vi) zona pellucida. Only 10^2^–10^3^ spermatozoa reach the cumulus–oocyte complex and even fewer bind and penetrate the zona pellucida [39]. During ART, natural sperm selection steps are bypassed, especially during ICSI, which bypasses all of these natural steps [5]. As a consequence, different effects may be obtained between IVF or ICSI when using sperm prepared by different methods. We thus compared whether the outcomes of conventional IVF and ICSI cycles differ when using sperm prepared by different methods (DGC vs. SU). The results showed no significant difference in the outcomes of either conventional IVF or ICSI cycles between the DGC and SU groups.

This study has several strengths. First, the sample size was large even after applying strict inclusion and exclusion criteria, which has strengthened the reliability of our results. Second, the main outcome of this study was the cLBR per aspiration, which reflects the effect of sperm quality on the overall embryo quality and development potential. Third, a series of potential confounders were adjusted using PS matching and generalized linear or logistic models to avoid bias. However, several limitations of our study remain. First, the retrospective nature of our study suggests an inherent risk of bias, despite adjusting for a series of potential confounders. Second, even if the sample size was large, it was insufficient for robust subgroup analyses. Third, the generality of the conclusion from this study is limited by the single-center design, as the varying laboratory and clinical procedures followed across different centers might affect the ART outcomes.

## Conclusions

To our knowledge, this is the largest study to compare the outcomes of IVF/ICSI cycles when using sperm prepared by DGC vs. SU, the two most widely used sperm preparation methods. We found that sperm prepared by these two methods resulted in similar cLBRs. Further study is warranted to compare the effects of these sperm preparation methods on IVF/ICSI outcomes when using sperm from subgroups such as men with poor semen parameters or high SDF.

## Supplementary Information

**Additional file 1: Supplementary Table 1.** Comparisons of outcomes between cycles preparing sperm with DGC and SU in cycles with different male age.

## Data Availability

The datasets used and/or analyzed during the current study are available from the corresponding author on reasonable request.

## Abbreviations

ART: Assisted reproductive technology
IVF: In vitro fertilization
ICSI: Intracytoplasmic sperm injection
SU: Swim-up
DGC: Density gradient centrifugation
WHO: World Health Organization
PR: Progressive motility rate
SDF: Sperm DNA fragmentation
CPR: Clinical pregnancy rate
OR: Odds ratio
CI: Confidence interval
PS: Propensity score
cLBR: Cumulative live birth rate
ET: Embryo transfer
BMI: Body mass index
AMH: Anti-Mullerian hormone
