# Supplementary material for: Cumulative live birth rates after IVF/ICSI cycles with sperm prepared by density gradient centrifugation vs. swim-up: a retrospective study using a propensity score-matching analysis
Source: Reprod Biol Endocrinol. 2022 Mar 31;20:60. doi: 10.1186/s12958-022-00933-2 (PMC8969370; doi:10.1186/s12958-022-00933-2)
Supplement: Supplementary file 1 — Additional file 1: Supplementary Table 1. Comparisons of outcomes between cycles preparing sperm with DGC and SU in cycles with different male age. [file 12958_2022_933_MOESM1_ESM.docx]

| Supplementary table 1 Comparisons of outcomes between cycles preparing sperm with DGC and SU in cycles with different male age. | | | | |
| --- | --- | --- | --- | --- |
| Outcomes | <=30 | 31-35 | 36-40 | >40 |
| No. of cycles |  |  |  |  |
| DGC | 241 | 241 | 149 | 88 |
| SU | 225 | 258 | 149 | 87 |
| Recovery rate, % | -0.017 (-0.093–0.060)^a^ | -0.011 (-0.084–0.062)^a^ | -0.023 (-0.134–0.089)^a^ | -0.026 (-0.179–0.126)^a^ |
| PR after preparation, % | -0.009 (-0.025–0.008)^a^ | 0.007 (-0.011–0.025)^a^ | 0 (-0.015–0.015)^a^ | -0.012 (-0.033–0.008)^a^ |
| Fertilization rate, % | 0.005 (-0.054–0.064)^a^ | 0.011 (-0.053–0.074)^a^ | 0.009 (-0.073 –0.091)^a^ | 0.056 (-0.073–0.185)^a^ |
| Good-quality embryo rate, % | 0.012 (-0.100–0.125)^a^ | 0.023 (-0.091–0.136)^a^ | 0.015 (-0.126–0.155)^a^ | 0.073 (-0.141–0.288)^a^ |
| Blastocyst formation rate, % | -0.038 (-0.144–0.068)^a^ | 0.099 (-0.044–1.852)^a^ | 0.081 (-0.127–0.289^a^ | -0.253 (-0.519–0.012)^a^ |
| cLBR, % (n/n) | 1.026 (0.653–1.611)^b^ | 1.211 (0.804–1.823)^b^ | 1.141 (0.649–2.004)^b^ | 1.110 (0.492–2.505)^b^ |
| LBR per transfer, % (n/n) | 1.029 (0.742–1.426)^b^ | 1.260 (0.919–1.728)^b^ | 1.060 (0.669–1.679)^b^ | 0.878 (0.452–1.706)^b^ |
| DGC, density gradient centrifugation; SU, swim-up; PR, progressive motility rate; cLBR, cumulative live birth rate | | | | |
| ^a^Adjusted β (95%CI), DGC vs. SU | | | | |
| ^b^Adjusted OR (95%CI), DGC vs. SU | | | | |
